# Systematic Review of Prognostic Gene Signature in Gastric Cancer Patients

**DOI:** 10.3389/fbioe.2020.00805

**Published:** 2020-07-31

**Authors:** Longxiang Xie, Linghao Cai, Fei Wang, Lu Zhang, Qiang Wang, Xiangqian Guo

**Affiliations:** Department of Preventive Medicine, Institute of Biomedical Informatics, Cell Signal Transduction Laboratory, Bioinformatics Center, Henan Provincial Engineering Center for Tumor Molecular Medicine, School of Software, School of Basic Medical Sciences, Henan University, Kaifeng, China

**Keywords:** gene signature, gastric cancer, overall survival, prognostic biomarker, TCGA, GEO

## Abstract

Gastric cancer (GC) is the second leading cause of cancer mortality and remains the fourth common cancer worldwide. The effective and feasible methods for predicting the possible outcomes for GC patients are still lacking. While genetic profiling might be suitable in some way, the application of gene expression signatures has been show to be a robust tool. Here, by performing a comprehensive search in PubMed, we provided an up-to-date summary of 39 prognostic gene signatures for GC patients, and described the processing procedure of the selection, calculation and construction of gene signature. We also reviewed current web tools including PROGgene and SurvExpress that can be used to analyze the prognostic value of multiple genes for GC. This review will aid in comprehensive understanding of the current prognostic gene signatures to accurately predict the outcome of GC patients, and may guide the future clinical management when the reliability of these signatures is validated in clinics.

## Introduction

Gastric cancer (GC) is one of the leading causes of cancer-related death in many countries. In China, GC is identified as the second-leading risk for cancer-related lethality, ranking the second in frequent malignancy for male and the third for female (Chen et al., 2016). Due to poor diet habits, the *Helicobacter pylori* (Hp) infection and insufficient early endoscopic screening techniques, the GC incidence and mortality rates remain highin China (Li and Kaminishi, 2009). It is estimated that there are 221,478 GC patient deaths in China every year, roughly half of the world's gastric cancer deaths in 2012 (Strong et al., 2015). Nowadays outcomes of GC patients are still undesirable poor even with the advancement in surgery, chemotherapy, and radiotherapy (Peng et al., 2018), the overall 5-year GC survival rate is below 30% (Miller et al., 2016). However, the effective methods for predicting the outcome for the purpose of timely appropriate intervention for GC patients are still lacking (Yin et al., 2013). Thus, there is high clincial demand on valid biomarkers to assess in advance patient prognosis and tailor patient management (Yu et al., 2019).

Several single molecules have been reported to be related with GC patients' outcomes and peritoneal relapse (Taniguchi et al., 1998; Ishii et al., 2000). For example, the over-expressed HER2 gene was found to be associated with the lymph gland metastasis of GC (Hecht et al., 2016). P53 was proved to be an unfavorable biomarker of GC (Wang et al., 2016). However, the single biomarker applied in prognosis is less robust relative than the multiple biomarker-based models (He and Zuo, 2019). Many studies have demonstrated that signatures with an optimal combination of several candidate biomarkers could note worthily improve the predictive accuracy (Hu et al., 2019). Hence, gene signatures comprised of multiple genes are developed to strengthen the ability in predicting prognosis of cancer patients. Several gene signatures have been constructed to accurately predict the GC prognosis (Cho et al., 2011; Wang et al., 2016, 2017a; Hou et al., 2017; Lee et al., 2017). In 2005, Chen et al. developed a prognostic model using three genes based on gene expression profiles of primary GC tumor samples and adjacent mucosas (Chen et al., 2005). In 2007, Marchet et al. constructed a model with three genes to predict the lymph node involvement of cancer cells in a cohort of 32 primary gastric carcinoma patients (Marchet et al., 2007). Consequently, the goal of our current study was to perform a comprehensive systematic review for published GC prognostic signatures derived from the genome-wide studies. In this study, we carried out a systematic review of reported prognostic signatures for GC, and identified 39 gastric cancer prognostic signatures. We also summarized 3 universal strategies of signature selections, calculations and constructions. Furthermore, the web tools for the prognosis assessment of gene signatures in GC were introduced and discussed.

## Materials and Methods

### Selection Criteria of Studies

**Figure 1:**
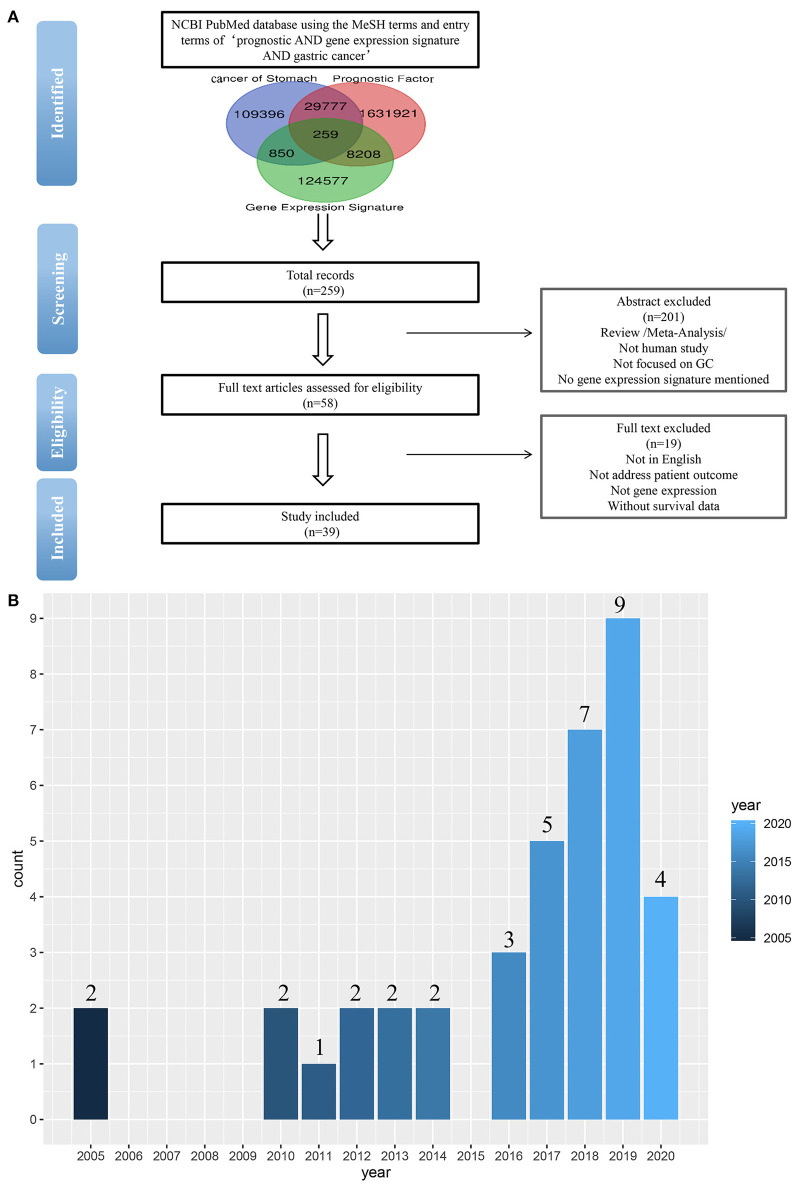
**(A)** Flow-chart diagram of literature collection (*n* = number of records). **(B)** The 39 studies published between 2005 and 2020.

To identify the published gastric cancer prognosis signatures, we firstly searched in NCBI PubMed database using both MeSH terms and entry terms of “prognostic AND gene expression signature AND gastric cancer.” We also checked recent reports and reviews on this topic. In this study, we considered and selected signatures that were derived from mRNA expression profiling studies and were proven to be related to patients' survival outcomes by independent validation (Figure 1A).

As a result of our search, a total of 259 studies were obtained by removing the duplication. Then we excluded some studies rigorously according to our criteria. The criteria of exclusion were as follows: (i) Review/Meta-Analysis; (ii) No human study/Not focused on GC; (iii) No gene-expression signature mentioned /Not address patient outcome; (iv) Without survival data. Finally, 39 signatures were collected from 39 studies published between 2005 and 2020 (Figure 1B).

### Statistical Collection

Two distinct survival association metrics were applied in evaluation of each prognostic model: (i) Hazard ratios (HRs) estimated by the Cox proportional-hazards regression model; (ii) The time-dependent receiver-operating characteristics (ROC) curves. Related information was listed in Table 1. We also provide the name and number of genes in each signature in Table 1 to facilitate the selection of the signatures for potential clinical application.

**Table 1:** Prognostic GC gene signatures collected in this review.

**Authors**	**Number of genes**	**Gene name**	**Survival event**	**Outcome**	**HR**	**AUC**	**Independent verification Cox**	**Method**	**PubMed ID**	**Strategy to develop signatures**
Jiang et al.	16	HSPA1A, HSPA1B, HSPA5, MICB, PSMC3, TAP2, KIAA0368, RBP1, APOD, VDR, PPP3R1, IL11RA, LGR4, NRP1, PLCG1, GZMB	OS	Unfavorable	3.9 (2.78–5.47)	/	/	Bioinformatics	31277152	II
Dai et al.	13	DCLK1, FLRT2, MCC, PRICKLE1, RIMS1, SLC25A15, SLCO2A1, CDO1, GHR, CD109, SELP, UPK1B, CD36	OS	Unfavorable	1.34 (1.19–1.51)	/	/	Bioinformatics	30702489	I
Zhao et al.	5	MARCKS, CCNF, MAPK14, INCENP, CHAF1A	OS	Unfavorable	2.03 (1.452–2.841)	/	Yes	Bioinformatics	30238991	II
Liu et al.	9	TOP2A, COL1A1, COL1A2, NDC80, COL3A1, CDKN3, CEP55, TPX2, TIMP1	OS	Unfavorable	/	1 year-0.696 3 year-0.741 5 year-0.838	/	Bioinformatics	30065754	I
Wu et al.	3	IHH, PTCH1, SMO	OS	Unfavorable	1.73 (1.26–2.39)	/	/	Bioinformatics/IHC	30008928	II
Zhu et al.	8	CAPN13, CBR1, LOXL1, CWH43, RAB31, PEX11G, ZNF57, ACADS	DFS	Unfavorable	4.00(2.41–6.66)	3 year-0.73 5 year-0.78	Yes	Bioinformatics/qRT-PCR	29957874	I
Wang et al.	5	COBLL1, DDB1, BCL2L13, NCOA6, FBXL11	OS	Unfavorable	2.35 (1.24–5.06)*	/	Yes*	Bioinformatics/qRT-PCR	23912700	I
Lee et al.	8	LAMP5, CDC25B, CDK1, CLIP4, LTB4R2, MATN3, NOX4, TFDP1	5 year-DFS	Unfavorable	3.16*	/	/	Bioinformatics	24598828	I
Pasini et al.	3	OLR1, CXCL11, ADAMDEC1	OS	Unfavorable	3.47 (1.23–5.06)	/	Yes	Bioinformatics/RT-PCR/IHC	24217965	I
Bauer et al.	3	NOTCH2, GSK3B, CTNNB1	1/2/3/5 year -OS	Favorable^+^	/	/	/	RT-PCR /IHC	22970250	II
Hou et al.	11	TRPC1, SGCETNFRSF11A, LRRN1,HLF, CYS1, PPP1R14A, NOVNBEA, CES1, RGN	OS	Unfavorable	/	0.769	Yes	Bioinformatics	28035468	I
Wang et al.	9	NR1I2, LGALSL, C1ORF198, CST2, LAMP5, FOXS1, CES1P1, MMP7, COL8A1	OS	Unfavorable	/	5 year-0.741	Yes	Bioinformatics	29088749	I
Peng et al.	12	ACOT7, CES1, IPMK, NES, PBX3, TMEM245, MIR6756, RAB11FIP4, RBPMS2, RPS27L, TPMT, TNFRSF11A	OS	Unfavorable	6.086	5 year-0.820	Yes	bioinformatics	29282891	I
Li et al.	4	KCNE2, PRPF3, KCNE2, API5	OS	Unfavorable	2.4	/	Yes	Bioinformatics	26840027	I
Cho et al.	6	CTNNB1, EXOSC3, TOP2A, LBA1, LZTR1, CCL5	RFS	Unfavorable	2.587	/	Yes	Bioinformatics/PCR	21447720	I
Wang et al.	25	APAF1, NCOA7, XAF1, IFITM1, EGFR, MMP7, MET, ERBB2, CDK1, CDK6, SRC, IGF1R, CDK4, KDR, FADD, EPHB2, PDCD5, MARCKS, GZF1, UBA2, MMP2, DYRK2, B3GALT6,TBP, PGK 1	OS	Unfavorable	6.248 (2.320–16.826)	/	Yes	Bioinformatics	28790411	I
Kuang et al.	11	CXCR5, CXCL13, CXCR1, CXCR2, CXCR4, ADCY7, P2RY13, BDKRB1, FPR2, RGS4, C5AR1	OS	Unfavorable	/	/	/	Clique Percolation/PCR/bioinformatics/ Sample collection/ RNA isolation	28798410	II
Fornaro et al.	3	CACNA-1G, CACNA-1H, CACNA-1I	OS	Unfavorable	1.75 (1.47–2.09)	/	Yes	Bioinformatics	28846697	III
Takeno et al.	22	RNF186,ZBTB1, RPSA, LMBR1, RPSA, STRBP, MC5R, C10orf95, pro1048, FIBP, ZIC3, WDR48, CKB, TXN, CTF1, Hypothetical gene supported by BC042812, GRPEL1, LTBP3, CHCHD3, CYP2W1, GDNF, PROK1	RFS	Unfavorable	6.37	/	Yes	Bioinformatics	20012501	I
Wang et al.	53	TUBB, GABBR1, KAT2A, FHOD1, CBFB, TNFAIP2, SMS, CAPRIN1, PTGS1, ECHDC2, GART, FGFR4, BAZ1A, CXCL10, ETFDH, SLC12A9, CEP55, LRRC41, PGRMC2, TGS1, CXCL1, LIMK1, LAMC2, ENC1, ADNP, ABCE1, CHORDC1, P4HA1, APOE, INHBA, OSMR, ATP13A3, APOC1, TCERG1, CCT6A, ALDH6A1, KLF4, SCNN1B, BCAR3, MMP14, PRC1, PNO1, ADH1C, COL6A3, SLC20A1, CCT2, PDP1, NOL8, EPHB4, MCM2, CPXM1, NCL, PRR7	OS	Unfavorable	/	/	Yes	Bioinformatics	27419373	I
Yin et al.	74	AR, CA2, COL1A1, COL1A2, COL3A1, COL5A1, COMP, KAL1, TGFBI, CTSK, NOTCH3, TWIST1, COL10A1, FCGR1A, SPP1, IGF2, ADH7, ATP4A, MGP, ACTN1, CA9, CHGA, ESRRG, BGN, COL8A1, COL15A1, EDNRA, F2R, FN1, INHBA, OLR1, PGF, SFRP4, TFF1, THBS2, THBS4, CHST1, TNFSF11, LIPF, CDC25B, FAP, MMP2, SPOCK1, MMP14, PLA2G7, GIF, PSCA, TUBB3, CCL7, THY1, PRRX1, TNFAIP6, TRIO, SPON2, SULF1, LEF1, NOX4, NOTCH1, NRP2, SMPD3, KIAA1199, KRT20, GKN1, PLXDC1, AMHR2, DMRT3, CIDEC, DMRTA1, FOXL2, COL18A1, FNDC1, CTHRC1, GKN2	OS	Favorable^#^	/	0.565	/	Bioinformatics/RT-PCR	24312559	I
Masaaki et al.	29	MRPS17, TRADD, QARS, MGC14141, BRD1, ODC1, POLRMT, NQO1, MACF1, PER2, DNAJA3, EPS8L3, AK056156, AK125944, LSM2, BC003075, AF040105, AC137055, ERBB2, DECR2, PTGS1, BC037493, GPI, FLJ13855, GBAS, CTSD, LYAR, UBXD1, liprin β2	RFS	Unfavorable	/	/	/	Clinical samples/ATAC-PCR/bioinformatics	15645432	I
Chen et al.	3	CD36, SLAM, PIM-1	OS	Unfavorable	/	/	Yes	Bioinformatics/ RT-PCR/ clinical samples	16145069	I
Xu et al.	4	ITGB1, PDGFB, THBS1, TWIST1	OS	Unfavorable	/	/	Yes	Bioinformatics/qRT-PCR/clinical samples	20005068	II
Kim et al.	3	MYC, EGFR, FGFR2	OS	Unfavorable	1.316	/	Yes	Bioinformatics/qRT-PCR/clinical samples	21173787	I
Peng et al.	6	CLEC11A, NRP2, TPM2, ANGPTL2, FGF7, FABP4, PODN	OS	Unfavorable	1.93 (1.42–2.62)*	/	Yes	Bioinformatics	32352015	II
Bai et al.	6	NPY, MICU3, TUBB6, RHO, MYO1A	OS	Unfavorable	2.995 (1.936-4.635)*	1 year-0.79 3 year-0.751 5 year-0.746	Yes	Bioinformatics	32174791	II
Guan et al.	10	HBB, C4orf48, MANEAL, CXCL3, TRIM31, TMEM200A, SERPINE1, F5, NOXO1, DKK1	OS/RFS	Unfavorable	/	OS-0.926 RFS-0.851	Yes	Bioinformatics	31966067	I
Jiang et al.	10	AKAP12, ANGPTL1, CYS1, MLLT11, NAV3, NBEA, NOV, PTN, TUSC3, ZSCAN18	OS/RFS	Unfavorable	OS-4.744 (2.988–7.352) RFS-11.36 (8.446–17.42)	0.898	Yes	Bioinformatics	31939629	I
Song et al.	5	FRMD7, FLJ16779, PRR20A, SLC7A2, SLC22A16	OS	Unfavorable	1.0559 (0.9995–1.1155)*	/	Yes	Bioinformatics	31422414	I
Yu et al.	4	NDRG1, NDRG2, NDRG3, NDRG4	OS	Unfavorable	1.76 (1.20–2.59)	0.679	/	Bioinformatics	31384305	III
Hu et al.	6	TMEM132C, PCOLCE2, UPK1B, PM20D1, SLITRK2	OS	Favorable	0.457 (0.318–0.658)	1 year-0.604 3 year-0.534 5 year-0.508	Yes	Bioinformatics	31247467	II
Chang et al.	5	KDM3A, P4HA1, ASPH, PLOD1, PLOD2	OS	Unfavorable	1.725 (1.142–2.605)	/	/	Bioinformatics	31036064	III
Chang et al.	16	WNT2, WNT3, WNT3A, WNT10B, FZD2, FZD6, FZD10, DVL3, WISP1, TBL1XR1, RUVBL1, MYC, CCND1, CAMK2B, RAC3, PRKCG	OS	Unfavorable	1.457	0.754	Yes	Bioinformatics	31028732	II
Smyth et al.	7	CDH1, ELOVL5, EGFR, PIP5K1B, FGF1, CD44v8. 10, TBCEL	OS	Unfavorable	5.1 (2.8–9.2)	/	/	Bioinformatics	30481267	I
Wang et al.	7	FBN1, MMP1, PLAU, SPARC, COL1A2, COL2A1, ATP4A	OS	Unfavorable	0.816	/	Yes	Bioinformatics	30133128	I
Yuzhalin et al.	9	Col11a1, SPP1, MFAP2, Col10a1, Col1a1, BGN, COMP, AGRN, MXRA5	OS/DFS	Unfavorable	1.59 (1.01–2.31)	/	/	Bioinformatics	29360819	II
Zhao et al.	17	SLC43A2, FAU, DAB2, COL5A1, ZCCHC2, ISY1, WDR1, DOCK10, C9orf142, SH3BP4, MRPS16, ALDH2, UBE2H, MAEA, CD58, CITED2, BNIP3L	OS	Unfavorable	/	/	/	Bioinformatics	26774142	I
Liu et al.	2	CACNB2, MEF2C	OS	Unfavorable	/	0.78933	/	Bioinformatics	31308482	I

HR from validation group is marked with *; Favorable+ means patients with GSK3B^high^, CTNNB1^high^ and NOTCH2^low^gene expression pattern; Favorable^#^ means patients with a 74-gene expression correlation coefficient morethan-0.007; / means not found.

## Results

**Figure 2:**
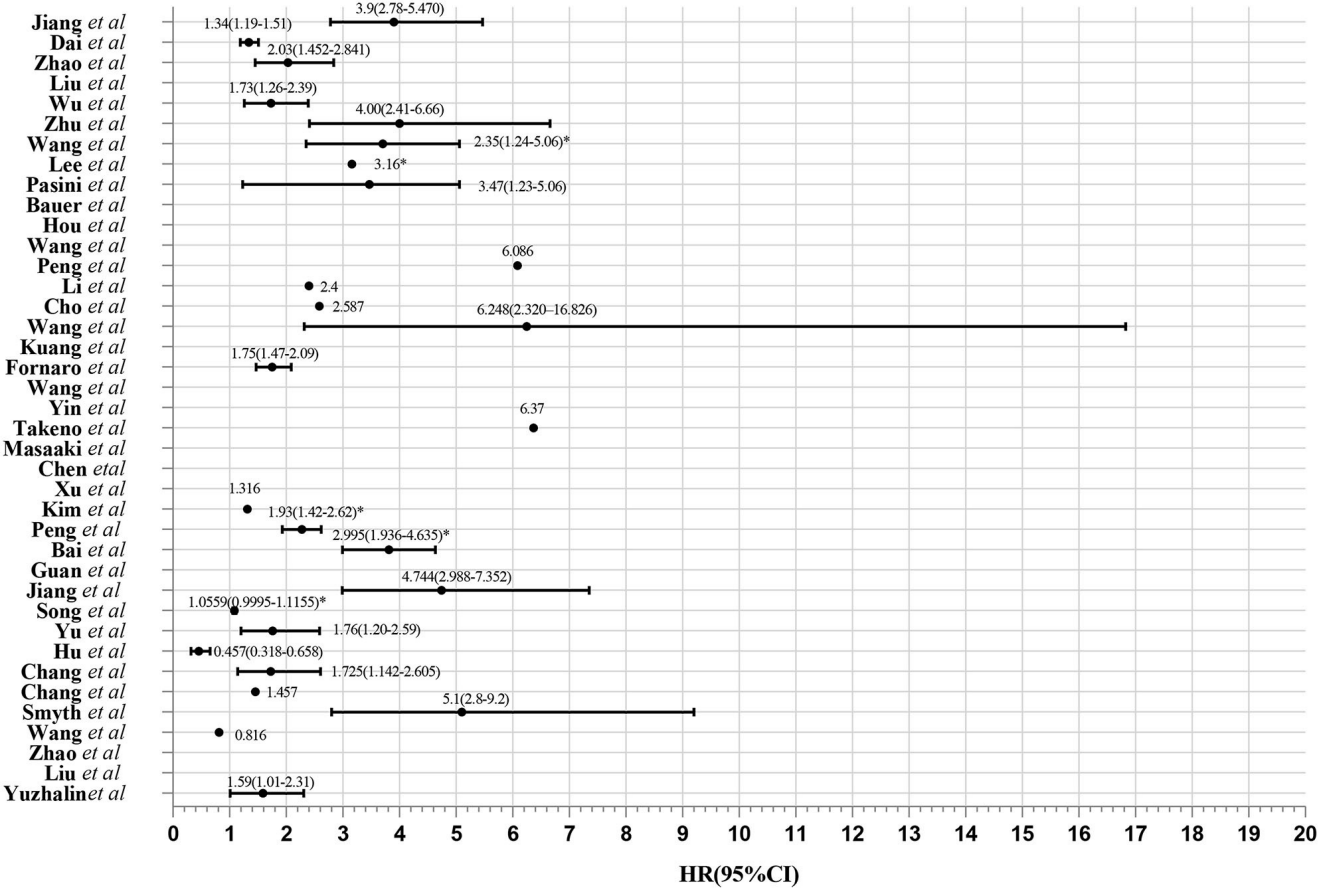
Evaluation of 39 gene expression signatures via Meta-analysis. HR from validation group is marked with *.

Elaborate search results of prognostic signatures in GC were shown in Table 1 and Figure 2 (Chen et al., 2005; Motoori et al., 2005; Xu et al., 2009; Takeno et al., 2010; Cho et al., 2011; Bauer et al., 2012; Kim et al., 2012; Wang et al., 2013, 2017b, 2018; Lee et al., 2014; Pasini et al., 2014; Li et al., 2016; Zhao et al., 2016, 2019; Hou et al., 2017; Kuang et al., 2017; Lafrenie et al., 2017; Liu et al., 2018, 2019; Peng et al., 2018, 2020; Smyth et al., 2018; Wu et al., 2018; Yuzhalin et al., 2018; Chang and Lai, 2019; Chang et al., 2019; Dai et al., 2019; Jiang et al., 2019, 2020; Song et al., 2019; Bai et al., 2020; Guan et al., 2020). Briefly, we got 39 literatures in NCBI PubMed Database following the above procedure (Figure 1). In total, 39 published gastric cancer prognostic signatures were identified, which were reported to be associated with patients' overall survival (OS), disease free survival (DFS) or recurrence-free survival (RFS) with 3 favorable signatures and 36 unfavorable signatures from 259 related articles in NCBI Pubmed. These signatures were constructed from diverse technological process, and we divided their construction methods into three strategies (I, II, and III), including basing on differentially expression genes (DEGs), specific cellular pathway and molecular family. For example, Jin et al. established a 13-mRNA (*DCLK1, FLRT2, MCC, PRICKLE1, RIMS1, SLC25A15, SLCO2A1, CDO1, GHR, CD109, SELP, UPK1B*, and *CD36*) signature by which patients with higher risk score showed worse survival in both TCGA and GEO datasets (Peng et al., 2018). Wu et al. constructed a three gene signature (*IHH, PTCH1*, and *SMO*) based on the hedgehog signaling pathway as the gene model which was shown to be a unfavorable independent prognostic indicator (Wu et al., 2018). Bauer et al. constructed a novel signature (*GSK3B, CTNNB1*, and *NOTCH2*), a strong predictor for favorable outcomes with *GSK3B*^high^, *CTNNB1*^high^ and *NOTCH2*^low^ (Bauer et al., 2012).

### The Selection and Calculation of Multi-Gene Signature

**Figure 3:**
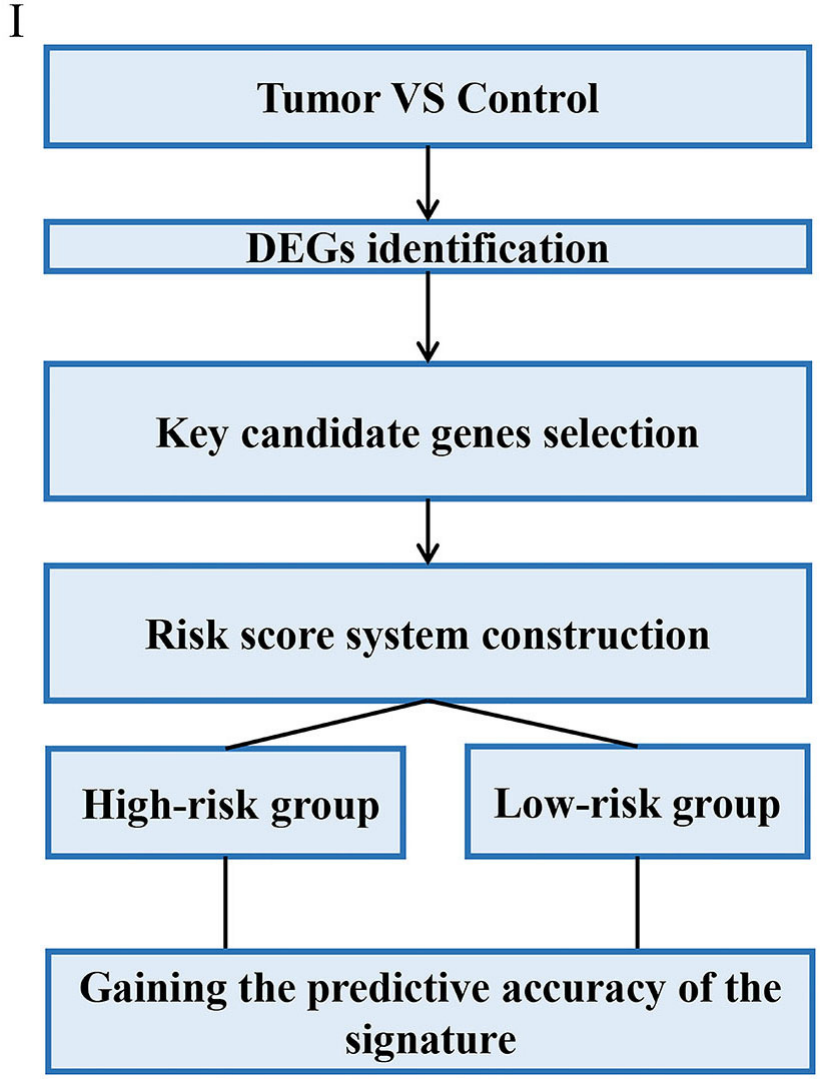
Strategy I relies on DEGs which are subject to signature construction.

**Figure 4:**
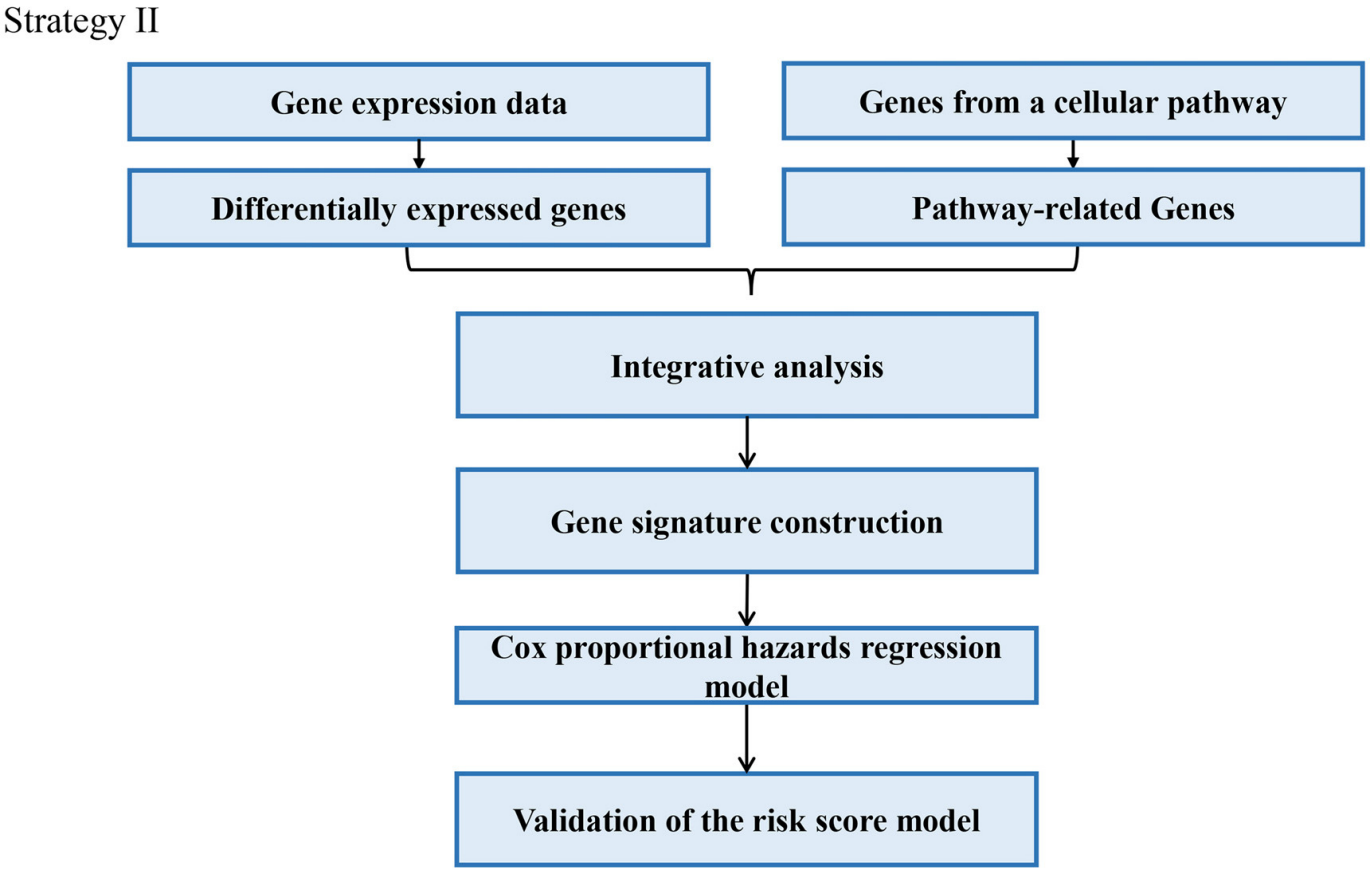
Strategy II utilizes genes from a typical pathway for signature construction.

**Figure 5:**
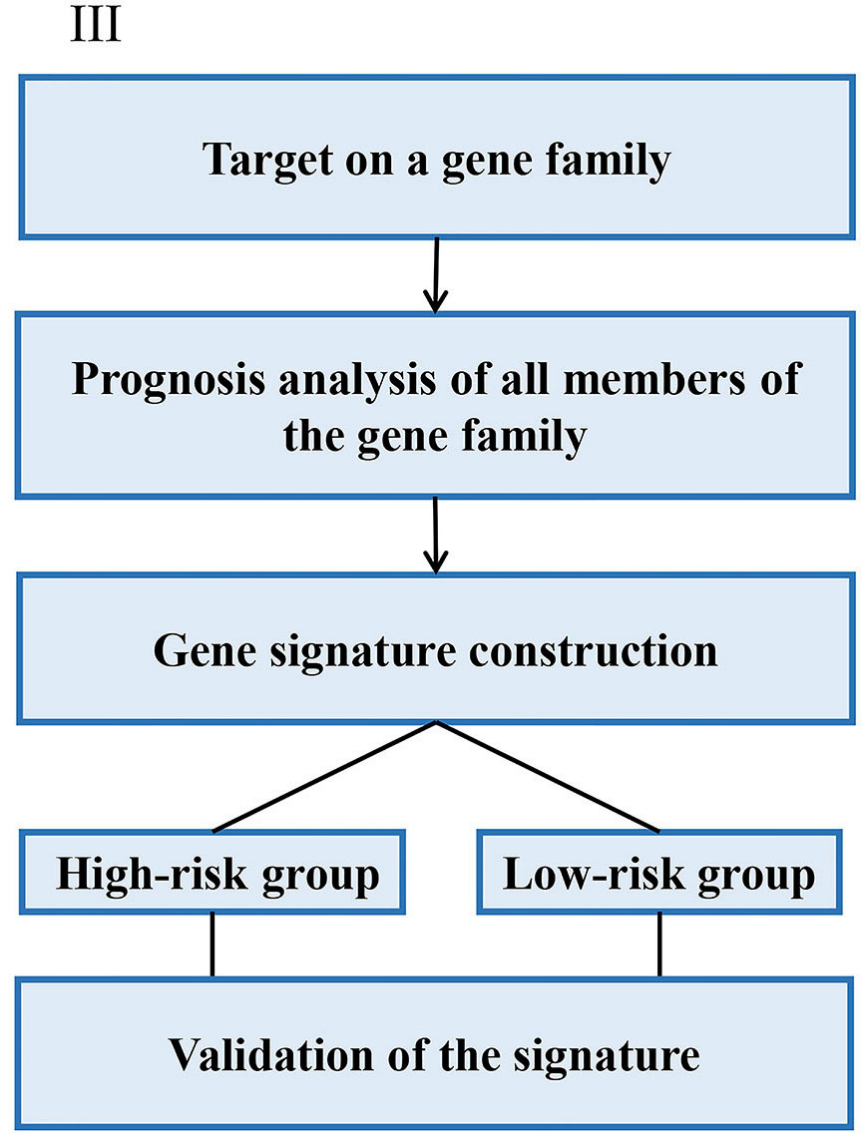
Strategy III focuses on the genes from a specific gene family for signature construction.

Through analyzing these 39 articles, we summarized three common strategies to construct gene signatures (Figures 3–5). The major difference in developing signatures is the source of potential genes. Strategy I refers to finding differentially expressed genes (DEGs) and selecting candidate genes through univariate and multivariate Cox regression analysis. Strategy II focuses on one gene group of typical pathways, like Hedgehog signaling pathway-associated genes, then filtering genes. Strategy III means to obtain potential genes from molecular family and construct signatures based on their subtypes.

#### Gene Signature Based on DEGs With High Prognostic Values

In Strategy I, authors established gene signatures based on DEGs with high prognostic values. Based on DEGs derived from Cancer Genome Atlas (TCGA), Dai et al. constructed a 13-mRNA signature to predict the prognosis of GC patients (Dai et al., 2019). Liu et al. identified nine hub genes and constructed a 9-gene signature based on gene expression profiling datasets by integrated bioinformatics analysis (Liu et al., 2018). Masaaki et al. selected only genes specifically expressed in gastric tissues from clinical samples and established a 29-gene signature (Motoori et al., 2005). The main steps of the workflow of strategy I were as follows: (i) Identification of DEGs associated with GC survival; (ii) Selection of key candidate mRNAs for the validation; (iii) Risk score model construction; (iv) Validation of risk score model. The overall process is presented in Figure 3.

For example, Liu et al. identified nine hub genes and constructed a nine-gene signature based on gene expression profiling datasets by integrated bioinformatics analysis (Liu et al., 2018). They firstly screened differentially expressed genes (DEGs) using microarray and RNA sequencing data and conducted certain integrated analysis, including functional enrichment for identifying the potential key genes involving the pathogenesis and prognosis of GC. Then these key genes that significantly correlated with patients' survival were regarded as candidate prognostic genes by univariate and multivariate Cox analysis. Finally, a prognostic gene signature was developed according to a linear combination of gene expression values multiplied by a regression coefficient (β) accessed from the multivariate Cox proportional hazards regression model of each gene. The formula is as follows: Risk score = β1X1 + β2X2 + β3X3 +…βnXn (Liu et al., 2018). In order to validate the prognostic power of the signature, all the patients were divided into low- or high-risk groups according to the median prognostic score. Then a time-dependent ROC curve analysis was performed to calculate the predictive ability of the gene signature for clinical outcomes.

#### Signature Constructed With Prognosis Related Pathway

In this strategy, authors established gene signatures from a specific gene group from typical cellular pathway. Peng et al. identified an Immune-Based Prognostic Signature (Peng et al., 2020). Bai et al. constructed six-gene signature based on DNA methylation-driven genes (Bai et al., 2020). Hu et al. established a metastasis-related gene (DMGs) signature that predicting prognosis in patients with gastric cancer (Hu et al., 2019). Chang et al. identified a core Wnt signature of 16 genes associated with Wnt signaling (Chang and Lai, 2019). The main steps of the workflow of strategy II were as follows: (i) Identification of DEGs in GC; (ii) Identification of a typical pathway-related genes in GC; (iii) Genes selected in the previous step were integrated to constructed a gene signature; (iv) The Cox proportional hazards model was applied to test their association with overall survival; (v) Validation of the risk score model; The overall process is presented in Figure 4.

For example, Wu et al. identified and validated a Hedgehog (Hh) pathway-based 3-gene prognostic signature for gastric cancer (Wu et al., 2018). They first analyzed the prognostic values of 9 canonical Hh signaling pathway-associated genes for GC patients. Three members *IHH, PTCH1*, and *SMO* were identified to have significant prognostic value at cutoff values. Based on the established cutoff value, patients were divided into subgroups with high- or low-risk respectively, and univariate Cox proportional-hazards regression analysis was carried out to calculate the coefficient for each of the three Hh-associated biomarkers. Subsequently, the prognostic risk for each cancer case was scored by summing the coefficient-weighted expression of the IHH-PTCH1-SMO signature as follows: 3-gene signature score = (0.553^*^IHH value) + (0.457^*^PTCH1 value) + (0.411^*^SMO value) (Wu et al., 2018). To validate the prognostic Hh gene signature, another GEO dataset was used as validating data set. In particular, they also performed independent validation of the prognostic significance by immunohistochemistry (IHC).

#### Signature Construction With a Specific Gene Family

In the strategy III, authors assessed the prognostic value of a specific gene family with updated public resources and integrative bioinformatics analysis. Yu et al. investigated the biological and prognostic values of the NDRG family in GC (Yu et al., 2019). Chang et al. examined the prognostic significance of oxygen-sensing genes from the 2-oxoglutarate-dependent oxygenase family (Chang et al., 2019). The main steps of the strategy III were as follows: (i) Target on a specific gene family; (ii) Analyzing of prognostic values of the gene family with different clinic pathological features; (iii) Constructing a prognostic model; (iv) Validation of the prognostic model. The overall process is presented in Figure 5.

For example, the N-myc downstream-regulated gene (NDRG) family, NDRG1-4 has been involved in a wide spectrum of biological functions in multiple cancers. From this perspective, Yu et al. firstly investigated the mRNA of the NDRG family was investigated in The Cancer Genome Atlas (TCGA). For each individual in the GC data of TCGA, a prognostic risk score was computed based on the risk score equation based on the score value with an optimal cutoff. High/low-risk groups were determined by the algorithm of the prognostic risk score. Finally, the low-risk group displayed a significantly favorable survival outcome than the high-risk group (Yu et al., 2019).

### Web Tools for the Prognosis Assessment of Gene Signature in Gastric Cancer

With advances in high-throughput techniques, a big volume of omics data were generated by next generation sequencing and gene microarray platforms, and these data have been deposited into public databases and can be leveraged to identify prognostic markers in different cancer types. Bioinformaticians have developed a number of online web tools for prognosis analysis such as OSkirc (Xie et al., 2019), OSlms (Wang et al., 2019), OSacc (Xie et al., 2019), OSblca (Zhang et al., 2019), OSuvm (Wang et al., 2019), PROGgene (Goswami and Nakshatri, 2013), SurvExpress (Aguirre-Gamboa et al., 2013), and KM plotter (Györffy et al., 2010). However, only two web servers including PROGgene and SurvExpress can be used to analyze the prognostic value of multiple genes as a signature.

In 2013, Goswami et al. implemented PROG gene, a survival analysis web tool based on gene expression for multiple cancer types. In 2014, they presented the second version of PROG gene, PROG geneV2, which has several enhancements over the previous version (Goswami and Nakshatri, 2014). In the PROGgeneV2, users can create the KM plots for published/curated gene signatures. PROGgeneV2 encompassed nearly ten thousand published/curated gene signatures from Molecular signature database to its repository. Users can directly search the keywords of gene signatures, and the application will retrieve genes included in the gene signature from the Molecular Signature Database. At last, a combined plot using mean of the expression of all genes in the signature will be presented for the entire signature (Goswami and Nakshatri, 2014).

In 2013, Raul et al. established SurvExpress, a web tool that performs the prognostic analysis of biomarkers and risk assessment for pan-cancers (Aguirre-Gamboa et al., 2013). SurvExpress can perform the assessment of single/multi-gene biomarkers in cancer. The prognostic index (PI, also called the risk score) is commonly utilized to stratify risk groups. Two methods are applied to generate risk groups in SurvExpress. The first method generates the risk groups by splitting the ordered PI (higher values for higher risk) with the median. The second method generates risk groups using an optimization algorithm from the ordered PI. A log-rank test was analyzed along all values of the arranged PI for two groups, and then the algorithm chooses the split point where the *p*-value is minimum.

## Discussion

In this study, we provided a systematic review of prognostic gene signature in gastric cancer patients. The purpose of this study is to provide a gene list for further prospective clinical application, but not to restate the procedures and results of the original publications. Compared to previous studies, our study completed the following: (i) Performing a systematic literature search for GC prognostic signatures and yielding a comprehensive signature collection; (ii) Extracting three common strategies to construct novel gene signatures; (iii) Reviewing current web tools including PROGgene and SurvExpress that can be used to analyze the prognostic value of multiple genes for GC. It is also necessary to consider and integrate with other types of gene signatures for GC prognosis, such as miRNA and lncRNA signatures (Cheng, 2018; Chen et al., 2019). Although we have summarized three general strategies to construct gene signatures, other solutions should also be mentioned. For example, Lukas et al. used different gene expression patterns of *GSK3B, CTNNB1*, and *NOTCH2* as a risk score to instead of using an equation to make risk score quantified. *GSK3B*^high^, *CTNNB1*^high^, and *NOTCH2*^low^ was linked with better outcomes (Bauer et al., 2012).

Chang et al. identified two signatures each consisting of a 5 genes, Signature 1 (*KDM8, KDM6B, P4HTM, ALKBH4, ALKBH7*) and signature 2 (*KDM3A, P4HA1, ASPH, PLOD1, PLOD2*), which can be used to predicting the OS in ten types of cancer patients (Chang et al., 2019). Yuzhalin et al. analyzed the extracellular matrix (ECM) genes significantly upregulated across a large cohort of patients with ovarian, lung, gastric and colon cancers and defined a nine-gene signature which was associated with poor prognosis in these cancer patients (Yuzhalin et al., 2018). Interestingly, these two literatures are both summarized in the strategy III. This might imply that the gene established by method III also has a good evaluation effect on the prognosis of other diverse types of cancer.

As well, we conducted a search in genes of 39 signatures to find the most overlapped gene. Three genes (*COL1A1, COL1A2, EGFR*) were used three times to construct signature in these articles, which indicated that they may be more powerful in GC prognosis and deserve to be noticed in GC prognostic gene signature construction in the future.

Notably, several drawbacks need to be discussed for these 39 gene signatures. For example, the limited number of clinical samples might affect there producibility of the prognostic signature, including more independent datasets from a certain or different race/ethnicity for cross-validation would improve the reliability of signature. Since GC incidences and survival outcomes differ significantly between Western and Asian countries (Macdonald, 2011). Zhu et al. found that their signature built based on Chinese patients is hard to be validated in the patients from other areas (Zhu et al., 2018). General signature research would also be limited by its retrospective study. The absent prospective study leads to low authenticity and acceptance of signatures in clinics. In a word, the combination of risk score and prospective randomized trials is of great necessities, in the hope that the true relevance of the risk score could be validated in the future study.

## Data Availability Statement

All datasets generated for this study are included in the article/Supplementary Material.

## Author Contributions

XG and LX: study concept and design. LX, LC, FW, LZ, and QW: acquisition of data. QW, LX, LC, and FW: analysis and interpretation of data. LX, LC, FW, and XG: draft of the manuscript and critical revision of the manuscript for intellectual content. All authors contributed to the article and approved the submitted version.

## Conflict of Interest

The authors declare that the research was conducted in the absence of any commercial or financial relationships that could be construed as a potential conflict of interest.

## Supplementary Material

Figure S1 Forest plot of the risk ratio for rates of survival on 39 gene signatures in GC.
